# The Retrograde Policy of the Bath Guardians

**Published:** 1914-06-06

**Authors:** Samuel Craddock

**Affiliations:** Late Medical Officer of the Bath Workhouse. Summerdale, Greenway Lane, Bath


### THE EDITOR'S LETTER-BOX.

### The Retrograde Policy of the Bath Guardians.

*To the Editor of* The Hospital.

Sir,—Under the heading of "The Retrograde Policy of the Bath Guardians" I notice in a part of a paragraph, beginning "Worst of all" that the workhouse infirmary was in such bad repute amongst the sick poor that they refused to enter it, saying "They would rather die at home." I give my unqualified denial of such a condition, a statement which is an absolute travesty of the truth and a malicious slander not only upon the Guardians, but upon every officer, more or less, associated with the administration of the infirmary—viz., the master and matron, the late superintendent nurse, the chaplain, and myself as medical officer, a post I held for thirty-one years. I assert most unequivocally that I believe there is no infirmary or hospital in England where the patients are better nursed, better fed, and their general well-being more considered than at Bath. There was a time when things were different.

During the period I have held the appointment of medical officer my one and sole object has been ceaseless anxiety to do all I could to render the patients' lives under the trials of sickness as bearable as possible, and I may add that for many years the Guardians have been equally sympathetic. Of course, I know not who could have given you such a description of the Bath Infirmary, but whoever he was I can only imagine he was influenced by a malignity that stifled truth and an utter disregard of existing conditions, as well as of respect for those responsible for the well-being of the sick and dying.

I am aware there is a strong feeling about the building of a new infirmary which has possibly been accentuated by a feeling that workhouses are doomed. My advice to you is, send a member of your staff to visit the infirmary and report after questioning the patients, and I am confident when you get that report you will feel your correspondent has grossly misled you, and I am sure an *amende honorable* will be forthcoming from you.—Yours, etc.,

Samuel Craddock,

Late Medical Officer of the Bath Workhouse.

Summerdale, Greenway Lane, Bath, June 2, 1914.

[We shall hope to act on Dr. Craddock's suggestion at a convenient date; we have omitted the last brief paragraph of his letter on account of a reckless use of adjectives which the writer himself would probably regret to see in print.—Ed. The Hospital.]

---

*To the Editor of* The Hospital.

